# Possible Role of Cytomegalovirus in Gastric Cancer Development and Recurrent Macrolide-Resistant *Campylobacter jejuni* Infection in Common Variable Immunodeficiency: A Case Report and Literature Discussion

**DOI:** 10.3390/microorganisms12061078

**Published:** 2024-05-27

**Authors:** Irene Díaz-Alberola, Andrea Espuch-Oliver, Francisco Fernández-Segovia, Miguel Ángel López-Nevot

**Affiliations:** 1 Servicio de Análisis Clínicos e Inmunología, Hospital Universitario Virgen de las Nieves, 18014 Granada, Spain; 2 Instituto de Investigación Biosanitaria de Granada (ibs.GRANADA), 18016 Granada, Spain; 3 Servicio de Análisis Clínicos, Hospital Universitario de Torrecárdenas, 04009 Almeria, Spain; 4 Servicio de Anatomía Patológica, Hospital Universitario Clínico San Cecilio, 18016 Granada, Spain; 5 Departamento de Bioquímica, Biología Molecular e Inmunología III, University of Granada, 18012 Granada, Spain

**Keywords:** common variable immunodeficiency, CVID, infections, cytomegalovirus, *Campylobacter jejuni*, gastric cancer, case report

## Abstract

Common variable immunodeficiency (CVID) is the most common symptomatic immunodeficiency in adults. It comprises a group of syndromes whose etiology involves genetic, epigenetic, microbiota, and environmental factors. We present the case of a 46-year-old Caucasian male patient with CVID and an immune dysregulation phenotype. The particular elements of the case consisted of an atypical clinical course, which undoubtedly demonstrates the great variability of clinical manifestations that these types of patients can suffer from, including bacterial and viral infections, autoimmune phenomena, and neoplasia. Notably, the patient suffered from recurrent gastrointestinal infection with macrolide-resistant *Campylobacter jejuni* and gastroduodenal disease and viraemia by cytomegalovirus (CMV). In addition, CMV was postulated as the main pro-oncogenic factor contributing to the development of early-onset intestinal-type gastric adenocarcinoma, for which the patient underwent gastrectomy. The patient’s evolution was difficult, but finally, as a result of the multidisciplinary approach, clinical stabilization and improvement in his quality of life were achieved. Based on our brief literature review, this is the first reported case of this clinical complexity. Our experience could help with the management of future patients with CVID and may also update current epidemiological data on CVID.

## 1. Introduction

Common variable immunodeficiency (CVID) is the most common symptomatic primary immunodeficiency (PID) in adults, including in the group of predominant antibody deficiencies [1]. The immune dysregulation in these patients predisposes them not only to recurrent infections but also to autoimmune disease, lymphoproliferation, or malignancy, resulting in significant morbidity and mortality [2]. To date, only a small percentage of cases have a monogenic defect involving genes that participate in multiple signaling pathways required for proper antibody production [3]. Recently, it has become increasingly clear that the etiology follows a complex polygenic inheritance pattern in which genetics, epigenetics, and environmental factors interact [4].

Gastrointestinal involvement in patients with CVID is very common and diverse, including both non-infectious and infectious diseases [5]. The most common gastrointestinal infections are caused by *Giardia*, *Campylobacter*, *Salmonella*, cytomegalovirus (CMV), and, more recently, *Norovirus* [6,7]. Gastrointestinal infection with *Campylobacter jejuni* (*C. jejuni*), a Gram-negative bacteria, is one of the most common causes of foodborne bacterial infection in the general population, typically being asymptomatic or presenting as a self-limiting diarrheal syndrome with spontaneous resolution [8]. However, in patients with CVID and hypogammaglobulinemia, gastrointestinal *C. jejuni* infection has been associated with recurrent, complicated, and prolonged infections, as well as other extraintestinal complications such as bacteremia [9]. While CMV enterocolitis and viremia have been reported in some patients with CVID and are known to contribute to their morbidity and mortality [10], the association of CMV with the development of intestinal-type gastric adenocarcinoma in a patient with CVID has not been previously reported. Patients with CVID are known to have a significantly higher risk of cancer than the general population. This includes gastric cancer, which is one of the most common cancers in CVID patients [11].

As far as we know, we report the first case of a Caucasian CVID patient with a severe immune dysregulation phenotype. This includes both infectious and non-infectious gastrointestinal involvement, such as recurrent macrolide-resistant *C. jejuni* infection and gastrointestinal CMV disease, as well as severe comorbidities such as bacteremia, autoimmune hemolytic anemia (AIHA), and early-onset intestinal-type gastric adenocarcinoma associated with CMV and *Helicobacter pylori* (*H. pylori*) infections. In this case, multidisciplinary interventions were required to stabilize the patient, ultimately resulting in the need for lifelong parenteral nutrition after gastrectomy.

## 2. Case Description

The patient is a 46-year-old Caucasian male who has suffered from chronic sinusitis and diarrhea since 1994. His family history includes ovarian cancer in his paternal aunt over the age of 50. He underwent splenectomy at the age of 34 due to a splenomegaly of more than 10 years of evolution after exclusion of splenic lymphoma. At the same age, he was diagnosed with CVID after admission for pneumococcal pneumonia complicated by pleural empyema and bacteremia, detecting immunoglobulin (Ig)G 50 mg/dL [540–1822], IgA 7 mg/dL [70–400], and IgM 4 mg/dL [22–240], in addition to total lymphopenia with CD8+ T cells 240/µL [270–930], CD4+ T cells 499/µL [440–1660], and B cells 71/µL [122–632]. Since then, he has been treated with intravenous Ig (IVIG) replacement at a dose of 0.6 mg/kg/3 weeks and antibiotic prophylaxis (sulfamethoxazole 800 mg and trimethoprim 160 mg).

**Figure 1:**
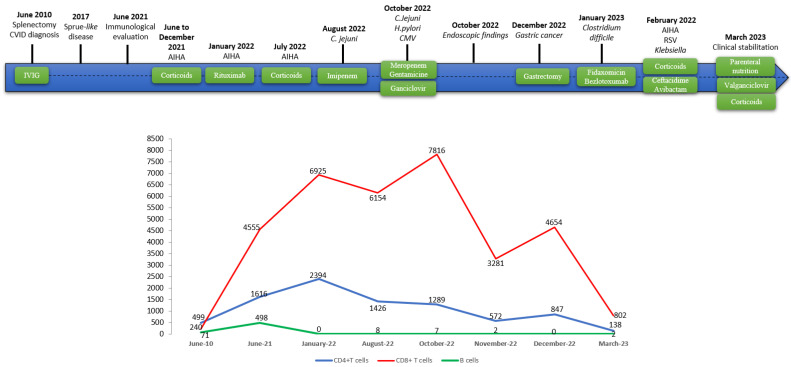
Timeline representation of the case. The timeline illustrates the different events in the course of the patient’s treatment, disease and lymphocyte count progression. CVID: common variable immunodeficiency; IVIG: intravenous immunoglobulin; AIHA: autoimmune hemolytic anemia; *C. jejuni*: *Campylobacter jejuni*; CMV: cytomegalovirus; RSV: respiratory syncytial virus.

The patient remained stable until the age of 41 years, when he required hospitalization for chronic mild diarrhea. Stool microbiological tests were negative, and upper gastrointestinal endoscopy (GE) revealed villous flattening and histology compatible with intraepithelial lymphocytosis. Chronic atrophic gastritis with intestinal metaplasia negative for *H. pylori* was detected, and celiac disease was suspected despite negative immunological tests. After one year on a gluten-free diet with no clinical response or change in the intestinal mucosa, celiac disease was ruled out, and he was diagnosed with sprue-like disease (Figure 1).

Given the chronic nature of the gastrointestinal manifestations and the poor adherence to immunoglobulin treatment, the patient was referred to the Immunology department in June 2021 for a reassessment of his immune status. IgG levels were 285 mg/dL, with undetectable levels of IgA, IgM, and IgE, so the IVIG replacement dose was modified to 0.6 mg/kg/2 weeks. In addition, B cells were normal (498/µL [122–632]), NK cells were reduced (78/μL [127–509]), and a CD3+ T lymphocytosis was detected at the expense of CD8+ T cells (4555/μL [270–930]) with normal CD4+ T cells (1616/μL [540–1660]), resulting in an inverted CD4/CD8 T ratio (0.35 [0.9–4.5]). Subsequent detailed examination of the CD8+ T cells by flow cytometry and molecular biological techniques revealed a polyclonal CD8+ T lymphocytosis, ruling out T lymphoma. The majority of CD8+ T cells expressed CD57+ (82%) and double-negative CD3+ T cells were increased in number (Supplementary Figure S1). In the B cell subpopulations, the absence of switched memory B cells [4–22%] and an increase in CD21^low^ B cells (28.3% [0.4–4.5%]) were notable. Complementary whole-exome sequencing (WES), filtered by a panel of 563 PID-associated genes, did not detect any pathogenic or likely pathogenic genetic variants that could explain the patient’s phenotype. However, three rare heterozygous variants of uncertain significance were identified (Supplementary Tables S1 and S2).

Between June 2021 and July 2022, the patient presented several episodes of AIHA with positive direct and indirect Coombs IgG tests. The first episode showed a good response to corticosteroids, but due to frequent relapses, treatment with rituximab was attempted but proved ineffective. Since then, the patient has been followed by the Hematology department and treated with low-dose maintenance corticosteroids (45 mg/day).

In August 2022, after a trip, the patient was admitted to the hospital with severe diarrhea of 15 stools per day, sometimes with blood, and abdominal pain. A stool culture revealed the presence of *C. jejuni*, which was initially treated with 1 g of oral azithromycin in a single dose and oral erythromycin for one week. The antibiogram showed that *C. jejuni* was macrolide-resistant, so the treatment was subsequently switched to intravenous imipenem for 2 weeks. Given the clinical improvement and the negative control stool culture, the patient was discharged. However, two weeks later, he was readmitted to the hospital with diarrhea (10–12 stools/day), weight loss (6 kg), asthenia, and bacteremia due to the same macrolide-resistant *C. jejuni* strain, which improved with meropenem and gentamicin. The presence of *H. pylori* fecal antigen was also detected, and the patient accepted its eradication after the current acute infection had ended. A new upper and lower GE was performed in October of the same year to assess gastrointestinal tissue damage. The endoscopic images showed multiple ulcers at the gastric level, excavated with a fibrinous background and regular contours, with normal mucosa. These ulcers were also seen in the duodenum and ileum, although smaller in size, but also some with a stellate appearance and multiple erythematous areas, in addition to an ulcer in the ileocecal valve. Biopsy samples were taken for histological and viral studies due to high suspicion of CMV or tumor but could not be evaluated due to poor quality. Instead, plasma viral DNA quantification was performed with a result of 17,500 UI/mL of CMV DNA copies. The patient was started on ganciclovir 50 mg/kg/12 h for 6 days.

**Figure 2:**
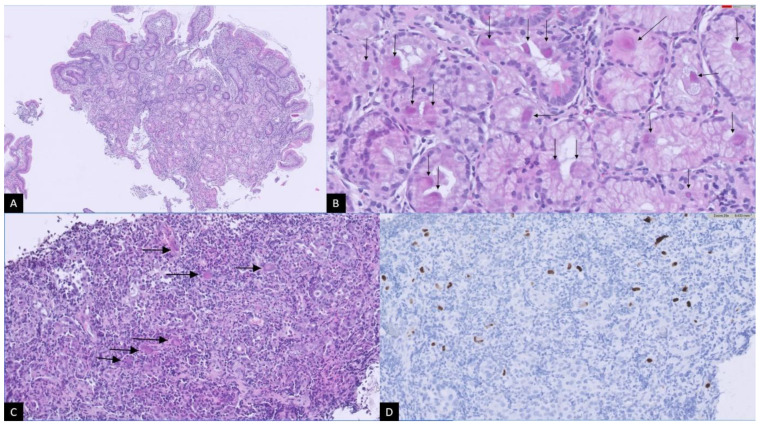
Histological findings: (**A**) Flattening of the duodenal villi and inflammatory infiltrate in lamina propria. Some epithelial cells with a more eosinophilic appearance were observed in Brunner’s glands. HE, 10×. (**B**) High magnification view of Brunner’s glands. Multiple epithelial cells of megalithic appearance with nuclear inclusions (Cowdry bodies) were seen (arrows). HE, 80×. (**C**) Infiltrating adenocarcinoma in the gastric wall. Abundant acute inflammatory background was observed. Cellular megalithic and nuclear inclusions in malignant epithelial cells (arrows). HE, 20×. (**D**) Immunohistochemistry for CMV in the stomach showed positivity in tumor epithelial cells and endothelial cells. IHQ 20×. HE: hematoxylin–eosin; CMV: cytomegalovirus.

A follow-up upper and lower GE was performed in December of the same year. On this occasion, a large antral lesion was observed, which obliterated all layers of the gastric wall and infiltrated the subserosa with minimal perilesional ascites and perigastric and mediastinal lymphadenopathy suggestive of neoplasia. Biopsies showed intestinal villous flattening and inflammatory infiltration of the lamina propria, in addition to an intestinal-type gastric adenocarcinoma. Cellular megalithic and nuclear inclusions were observed in the intestinal and gastric regions. Immunohistochemistry confirmed the presence of CMV in both regions, including at the gastric level in both tumor and endothelial cells (Figure 2), confirming the presence of CMV gastroduodenal disease and viremia.

PET-CT showed multiple small retroperitoneal, mediastinal, and diaphragmatic nodules with mild uptake (SUVmax = 1.2–2.8), and the final tumor stratification was cT3N1. After multidisciplinary discussion, the patient underwent total gastrectomy with D1+ lymphadenectomy in late December 2022. It was decided to add imipenem and delafloxacin for 5 days to prevent a possible recurrence of *C. jejuni* and oral valganciclovir 900 mg/12 h for 18 days to control CMV viremia. Subsequent CMV monitoring showed progressive reductions in viral load and T cell counts (CD4+ T cells 847/μL [540–1660] and CD8+ T cells 4654/μL [270–930]), and he was offered lifelong antiretroviral therapy. Given the patient’s refusal, prophylactic therapy with weekly CMV viral load monitoring was offered. In addition, because of the clinical stabilization and taking advantage of the hospital admission, it was decided to start *H. pylori* eradication treatment with 3 capsules of Pylera^®^ every 6 h for 4 days, which was completed with good tolerability. The patient was discharged with an undetectable CMV viral load and a normal absolute lymphocyte count (2780/μL [1100–4500]).

At the end of January 2023, the patient is readmitted because of diarrhea, fever, and abdominal discomfort. *Clostridium difficile* toxin was detected in the stool, along with new growth of *C. jejuni*. A watchful waiting policy was maintained for *C. jejuni*, but treatment was started with fidaxomicin (200 mg/12 h for 5 days, then 200 mg/48 h for 20 days) and a single dose of bezlotoxumab 650 mg intravenously. During this admission, he also had an episode of AIHA controlled with oral prednisone 100 mg/day, a respiratory syncytial virus (RSV) infection treated with mechanical ventilation, oxygen therapy, and bronchodilators, and bacteremia due to *Klebsiella pneumoniae* treated with ceftacidime/avibactam and then ceftriaxone 2 g IV for 7 days. At this point, fecal transplantation was considered, but the next stool culture was negative, and the colonoscopy was normal despite persistent mild diarrhea. After a 48-hour fast, the diarrhea resolved, and a multifactorial etiology (recurrent infections, gastrectomy, and dysbacteriosis) was suspected. Due to the patient’s high degree of malnutrition, parenteral nutrition was started. Two months later (March 2023), the patient was discharged with a prophylactic regimen of valganciclovir 450 mg/2 tablets/day, methylprednisone 60 mg/day, IVIG 0.6 mg/kg/2 weeks, and home parenteral nutrition. 

The patient is currently stable and continues to be closely monitored by the Gastroenterology, Hematology and Immunology departments, with a normal total lymphocyte count (1230/μL [1100–4500]) and persistent neutrophilia (17,770/μL [1500–7700]), with no infectious cause identified to date. 

## 3. Discussion

CVID is the most common symptomatic primary immunodeficiency in adults. With an estimated incidence of approximately 1 in 20,000, it is one of the most common immunodeficiencies encountered in clinical practice [12]. Depending on the clinical manifestation, patients with CVID can be classified into infection-only phenotypes or CVID with immune dysregulation (autoimmune complications, lymphadenopathy with splenomegaly, granulomatous disease, hepatopathy, or enteropathy) [13]. To our knowledge, this is the first report of a Caucasian CVID patient with a severe immune dysregulation phenotype, including infectious and non-infectious gastrointestinal involvement such as recurrent macrolide-resistant *C. jejuni* infection, severe comorbidities such as bacteremia, AIHA, and early-onset intestinal-type gastric adenocarcinoma associated with CMV and *H. pylori* infection.

*C. jejuni* is one of the most common causes of gastrointestinal infections in patients with CVID [14]. Such immunocompromised patients are more likely to develop severe complications after *C. jejuni* infection, to suffer from a chronic carrier status, or to have recurrent symptomatic episodes over years [13]. To date, the available literature is mainly limited to individual reports or series of CVID patients suffering from chronic or recurrent *C. jejuni* infection and/or complications such as bacteremia [9,15,16,17,18,19]. However, a cohort study of CVID patients with *Campylobacter* infection has recently been published [14]. Similar to the other previously reported cases, the patient presented with diarrhea, other gastrointestinal symptoms, and weight loss. Reduced or undetectable IgA and long-term average IgG levels below baseline are other typical features found [9,13,19]. IgA deficiency is considered one of the risk factors contributing to the susceptibility of CVID patients to gastrointestinal bacterial infections due to its key role in the intestinal mucosal defense [16]. Poor adherence to treatment and gastrointestinal protein loss probably contributed to the inadequate IgG levels. Decreased T cell levels have also been observed previously [14,19], suggesting the presence of T cell dysfunction in CVID patients. The patient’s immunodeficiency and the use of concomitant immunosuppression may also contribute to T cell depletion, increasing susceptibility to infection. As most *Campylobacter* spp. lack many of the classical virulence factors of gastrointestinal bacterial pathogens, the resulting intestinal pathology is likely to be due to the host immune response [20,21,22]. Severe comorbidities, and in particular *C. jejuni* bacteremia, are detected in <1% of patients [18], but are more likely to occur in immunocompromised patients [17]. The IgM deficiency may be critical for the failure of its bactericidal effect in the blood, as well as the previously described alteration in intestinal permeability, which contributes to bacterial translocation from the intestinal mucosa into the blood and *C. jejuni* bacteremia [23]. The existence of an interaction between the gut microbiota and the immune system, which is necessary to maintain immune homeostasis, is becoming increasingly clear [24]. Changes in the diversity of the gut microbiome have been observed in several studies in patients with CVID compared to healthy individuals [25], and are even associated with systemic inflammation and disease severity [26]. Undoubtedly, in our case, the multiple antibiotic therapies he received during his clinical course, as well as other therapies due to his autoimmune comorbidities, continued to favor dysbiosis and alteration of the intestinal barrier. A dysfunctional intestinal barrier promotes colonization by other microorganisms, such as *Clostridium difficile* (*C. difficile*), although its incidence has been shown to be low in this type of patient [5,27]. In such severe cases, fecal transplantation could be a potentially beneficial therapy to restore intestinal normality, which is currently only approved for refractory or recurrent *C. difficile* infection. However, several issues need to be addressed before fecal transplants can be used in patients with CVID. Other treatments that may be considered include personalized prebiotics or probiotics [23,24].

The subsequent multiple detections of *C. jejuni* in our case are consistent with pathogen persistence rather than reinfection, reflecting the significant difficulty in eradicating gastrointestinal pathogens in this type of patient. Macrolides are considered the first-line treatment for *C. jejuni* infection. However, macrolide-resistant strains have increased in recent years and are a major concern in developed countries [28]. In previous studies of CVID patients, antibiotic resistance was common, especially to macrolides and fluoroquinolones [14]. In our case, the failure of treatment with azithromycin and erythromycin led to a request for an antibiogram, which revealed the presence of a macrolide-resistant strain sensitive to imipenem and fosfomycin. Currently, an antibiotic treatment strategy for immunocompromised patients with recurrent *Campylobacter* infections is not established, although some workflow therapeutic strategies have been proposed [14]. In our case, we chose to maintain carbapenems, broad-spectrum antibiotics that are usually effective in multi-resistant cases and those complicated by bacteremia, together with gentamicin for a prolonged period. However, fosfomycin was also considered, as it has shown a safe and effective profile in these cases [29].

CMV is an encapsulated human beta-herpesvirus with a high prevalence in the adult population [30]. CMV infection is usually asymptomatic or mild in immunocompetent individuals, leading to lifelong persistent infection with periods of reactivation, and can cause more serious conditions in immunocompromised individuals [31]. There are few reported cases of CMV gastrointestinal disease [32,33] and viremia in patients with CVID [34,35]. CMV should be considered in the differential diagnosis of patients with CVID and gastrointestinal involvement, emphasizing not only impaired B cell immunity but also impaired T cell immunity. Molecular or immunohistochemical studies must be the main diagnostic tools, as serological studies are misleading in these patients [36]. In biopsied tissues, the diagnosis of CMV infection is classically based on the histopathological identification of virus-infected cells (viral cytopathic effect) on hematoxylin–eosin-stained slides and/or the detection of intranuclear CMV inclusions by immunohistochemical studies [37]. In our case, this finding was crucial in confirming CMV infection. The T lymphocyte population plays an important role in anti-viral immunity against CMV, in particular the CMV-specific CD8+ T cells, but also CD4+ T cells, by promoting the priming, expansion, and maintenance of these CD8+ CMV-specific T cells and by controlling episodes of viral reactivation [38]. A polyclonal expansion of CD8+ T cells with CD57+ expression, an exhaustion or senescence phenotype, has been previously described and associated with persistent or chronic CMV infection [35,39]. This immunological phenotype was detected in the patient since 2021, when he was referred to the Immunology department, so CMV infection was probably already present and partly responsible for the gastrointestinal manifestations but was not investigated. The lack of CMV-specific CD8+ T cells and normal CD4+ T cells in the patient could explain his clinical course, but we cannot perform this immunological study in depth. 

To the best of our knowledge, this is the first reported case of early-onset intestinal-type gastric adenocarcinoma, probably associated with the CMV infection in a patient with CVID. Only one case of early-onset moderately differentiated and gland-forming gastric adenocarcinoma with recurrent CMV infection has been reported in a South Asian female with CVID [40]. It is also more common in patients with immune dysregulation disorders such as CTLA haploinsufficiency or LRBA deficiency [41]. In our case, no pathogenic variant was detected in genes associated with primary immunodeficiencies, as is the case in most patients with CVID. Gastric cancer is the second most common type of cancer reported in CVID [42]. Chronic atrophic gastritis has been shown to be the strongest predictor of the onset of gastric adenocarcinoma [43] and, together with intestinal metaplasia, is a known risk factor for the development of intestinal-type gastric cancer [44]. Both conditions were present in the patient at the age of 41, when he was diagnosed with sprue-like disease. In addition, intraepithelial lymphocytosis in patients with CVID has previously been associated with an altered type I interferon response, which may explain an altered response to the CMV in this case [26]. CMV has previously been implicated in causing gastric ulcers [45,46]. Recently, it has been postulated as a possible oncogenic herpesvirus and a risk factor for the development of gastric cancer [47,48,49], although it is still a controversial issue for some authors [50]. We believe that in our reported case, CMV is one of the main drivers of gastric cancer due to its presence at the gastric level in tumor cells. After gastrectomy and antiretroviral treatment, the CMV viral load was undetectable and lymphocyte levels were normal, which would support our theory. *H. pylori* infection probably contributed synergistically to the patient’s inflammatory complications and gastric tumor transformation, following the Correa cascade carcinogenesis mechanism [51], previously reported in immunocompetent cases [52,53]. In addition, other factors such as the patient’s immunosuppression, viral immune escape mechanisms and other genetic and epigenetic factors present in the patient may have contributed [40].

Finally, patients with CVID may also suffer from autoimmunity disorders. AIHA is the second most common autoimmune cytopenia found in CVID patients, with a characteristic phenotype that includes decreased switched memory B cells and expansion of CD21^low^ B cells [54]. CD21^low^ cells are considered autoreactive cells and have also been associated with chronic immune activation and splenomegaly [55,56]. Indeed, splenomegaly is quite common in these patients and is associated with autoimmune features. Patients with CVID and splenomegaly usually have peripheral lymphopenia and decreased switched memory B cells. Splenectomy usually restores the absolute circulating lymphocytes, although B cell subpopulations don’t change [57]. All of this is consistent with the immunophenotype of our patient. Despite a splenectomy, the patient continued to have episodes of AIHA. Undoubtedly, the rituximab treatment used in his second AIHA relapse was inappropriate and favored hypogammaglobulinemia and persistent B cell lymphopenia, which in turn did not help with his subsequent bacterial infections. 

## 4. Conclusions

This case report undoubtedly demonstrates the great variability of manifestations that patients with CVID can present, including bacterial or viral infections, autoimmune phenomena, and the development of neoplasms. Therefore, a multidisciplinary approach is essential to improve their quality of life and adequately manage their comorbidities. Bacterial gastrointestinal infections can be difficult to eradicate and are sometimes associated with recurrences and more serious and atypical complications, such as macrolide-resistant *C. jejuni* bacteremia. Gastrointestinal viral infections, although less common, should also be considered in the differential diagnosis of CVID with gastrointestinal involvement, and their diagnosis should be confirmed by molecular diagnosis or immunohistochemistry, as serological studies would be false negatives. A CMV infection could be suspected in the presence of diarrhea with markedly exhausted CD8+ T expansion, and should also be considered as a possible pro-oncogenic virus. Given the increased risk of cancer in patients with CVID, early treatment of infections caused by pro-oncogenic gastrointestinal pathogens is essential, as is the establishment of an early standardized gastric screening protocol. Finally, the dysbiosis found in these patients may worsen their clinical course. Because of the clear link between the gut and the immune system, fecal transplantation should be considered in the future as a therapy for such severe cases.

## Data Availability

All data regarding the findings are available within the manuscript.

## Supplementary Materials

The following supporting information can be downloaded at https://www.mdpi.com/article/10.3390/microorganisms12061078/s1, Table S1: PID associated genes evaluated by WES; Table S2: Rare uncertain significance variants identified in the patient. The reference human genome used for the alignment phase was GRCh37/hg19. The American College of Medical Genetics and Genomics classification and GnomAD exome population frequency were based on the results consulted at march 2024; Figure S1: Immunophenotyping of T cells. Of the total number of lymphocytes, 94.3% are CD3+ T cells (represented with SSC-Linear and CD45+). Of these, an increase of CD8+ T cells (78% of the total CD3+) and 12.4% of double negative T cells (CD3+CD4-CD8-) were observed. Of the total CD8+ T cells, 82% expressed the CD57 marker.
