# Supplementary material for: Possible Role of Cytomegalovirus in Gastric Cancer Development and Recurrent Macrolide-Resistant Campylobacter jejuni Infection in Common Variable Immunodeficiency: A Case Report and Literature Discussion
Source: Microorganisms. 2024 May 27;12(6):1078. doi: 10.3390/microorganisms12061078 (PMC11205354; doi:10.3390/microorganisms12061078)

SUPPLEMENTARY MATERIAL

Table S1. PID associated genes evaluated by WES.

ABCB1, ACD, ACP5, ACTB, ADA, ADA2, ADAM17, ADAMTS13, ADAR, ADGRE2, AICDA, AIRE, AK2, ALPI, ALPK1, ANKRD26, AP1S3, AP3B1, AP3D1, APOL1, ARHGEF1, ARPC1B, ARPC5, ATAD3A, ATG16L1, ATG4A, ATM, ATP6AP1, B2M, BACH2, BANK1, BCL10, BCL11B, BLK, BLM, BLNK, BRCA1, BRCA2, BRIP1, BTK, BTNL2, C1QA, C1QB, C1QC, C1R, C1S, C2, C2orf69, C3, C4A, C4B, C5, C6, C7, C8A, C8B, C8G, C9, CARD11, CARD14, CARD8, CARD9, CARMIL2, CASP10, CASP8, CBLB, CCBE1, CCDC28B, CCDC88B, CCL2, CCR2, CCR5, CD19, CD209, CD22, CD247, CD27, CD28, CD36, CD3D, CD3E, CD3G, CD4, CD40, CD40LG, CD46, CD55, CD59, CD70, CD79A, CD79B, CD81, CD8A, CDC42, CDCA7, CEBPE, CFB, CFD, CFH, CFHR1, CFHR2, CFHR3, CFHR4, CFHR5, CFI, CFP, CFTR, CHD7, CHUK, CIB1, CIITA, CISH, CLCN7, CLEC7A, CLPB, COG6, COPA, COPG1, CORO1A, CR2, CRACR2A, CSF2RA, CSF2RB, CSF3R, CTC1, CTLA4, CTNNBL1, CTPS1, CTSC, CXCR2, CXCR4, CYBA, CYBB, CYBC1, CYCS, DBR1, DCLRE1C, DEF6, DGAT1, DGKE, DIAPH1, DKC1, DNAJC21, DNASE1, DNASE1L3, DNASE2, DNMT3B, DOCK2, DOCK8, DPP9, DSG1, EFL1, EGFR, ELANE, ELF4, EPCAM, EPG5, ERBIN, ERCC4, ERCC6L2, ETS1, ETV6, EXTL3, F12, FAAP24, FADD, FANCA, FANCB, FANCC, FANCD2, FANCE, FANCF, FANCG, FANCI, FANCL, FANCM, FAS, FASLG, FAT4, FCGR1A, FCGR2A, FCGR2B, FCGR3A, FCGR3B, FCHO1, FCN3, FERMT1, FERMT3, FLG, FNIP1, FOXN1, FOXP3, FPR1, FUT2, FYB1, G6PC3, G6PD, GATA1, GATA2, GFI1, GINS1, GINS4, GNAI2, GTF3A, GUCY2C, HAVCR2, HAX1, HCK, HELLS, HLA-DQB1, HLA-DRB1, HMOX1, HYOU1, ICAM1, ICOS, ICOSLG, IFIH1, IFNAR1, IFNAR2, IFNG, IFNGR1, IFNGR2, IGHM, IGKC, IGLL1, IKBKB, IKBKG, IKZF1, IKZF2, IKZF3, IKZF5, IL10, IL10RA, IL10RB, IL12B, IL12RB1, IL12RB2, IL17F, IL17RA, IL17RC, IL18BP, IL1RN, IL21, IL21R, IL23R, IL2RA, IL2RB, IL2RG, IL36RN, IL37, IL6, IL6R, IL6ST, IL7, IL7R, INAVA, INO80, IRAK1, IRAK4, IRF1, IRF2BP2, IRF3, IRF4, IRF5, IRF7, IRF8, IRF9, IRGM, ISG15, ITCH, ITGB2, ITK, ITPKB, JAGN1, JAK1, JAK2, JAK3, KDM6A, KMT2A, KMT2D, KRAS, LACC1, LAMTOR2, LAT, LCK, LCP2, LIG1, LIG4, LPIN2, LRBA, LRRC8A, LSM11, LYST, MAD2L2, MAGT1, MALT1, MAN2B1, MAN2B2, MAP1LC3B2, MAP3K14, MAPK8, MASP1, MASP2, MBL2, MC2R, MCM10, MCM4, MECOM, MED14, MEFV, MMACHC, MOGS, MPO, MRTFA, MS4A1, MSH6, MSN, MST1, MTHFD1, MVK, MYD88, MYH9, MYO5B, MYSM1, NBAS, NBN, NCF1, NCF2, NCF4, NCKAP1L, NCSTN, NEUROG3, NFAT5, NFE2L2, NFKB1, NFKB2, NFKBIA, NHEJ1, NHP2, NLRC4, NLRP1, NLRP12, NLRP3, NOD2, NOP10, NOS2, NRAS, NSMCE3, NUP214, OAS1, ORAI1, OSTM1, OTULIN, P2RX7, PALB2, PARN, PAX1, PDCD1, PDGFRA, PDLIM1, PEPD, PERCC1, PGM3, PHF11, PI4KA, PIK3CD, PIK3CG, PIK3R1, PLCG2, PLEKHM1, PLVAP, PMS2, PNP, POLA1, POLD1, POLD2, POLE, POLE2, POLR3A, POLR3C, POLR3F, POMP, POU2AF1, PRF1, PRKCD, PRKDC, PRKG1, PRPS1, PSEN1, PSENEN, PSMB10, PSMB4, PSMB8, PSMB9, PSMD12, PSMG2, PSTPIP1, PTEN, PTPN2, PTPN22, PTPRC, RAB27A, RAC2, RAD51, RAD51C, RAG1, RAG2, RANBP2, RASGRP1, RBCK1, RC3H1, RECQL4, REL, RELA, RELB, RFWD3, RFX5, RFXANK, RFXAP, RHOG, RHOH, RIPK1, RMRP, RNASEH2A, RNASEH2B, RNASEH2C, RNF168, RNF31, RNU4ATAC, RNU7-1, RORC, RPSA, RTEL1, SAMD9, SAMD9L, SAMHD1, SASH3, SBDS, SEC61A1, SEMA3E, SERPING1, SH2D1A, SH3BP2, SH3KBP1, SIAE, SKIV2L, SLC11A1, SLC26A3, SLC29A3, SLC35C1, SLC37A4, SLC39A7, SLC46A1, SLC7A7, SLC9A3, SLX4, SMARCAL1, SMARCD2, SNORA31, SNX10, SOCS1, SP110, SPATA5, SPI1, SPINK5, SPINT2, SPPL2A, SRC, SRP54, SRP72, STAT1, STAT2, STAT3, STAT4, STAT5B, STIM1, STING1, STK4, STN1, STX11, STXBP2, SYK, TAFAZZIN, TAP1, TAP2, TAPBP, TBK1, TBX1, TBX21, TCF3, TCIRG1, TCN2, TERC, TERT, TET2, TFRC, TGFB1, TGFBR1, TGFBR2, THBD, TICAM1, TINF2, TIRAP, TLR1, TLR2, TLR3, TLR5, TLR7, TLR8, TMC6, TMC8, TNF, TNFAIP3, TNFRSF11A, TNFRSF13B, TNFRSF13C, TNFRSF1A, TNFRSF4, TNFRSF9, TNFSF11, TNFSF12, TNFSF13, TNFSF15, TNFSF4, TOP2B, TP53, TPP2, TRAC, TRAF3, TRAF3IP2, TREX1, TRIM22, TRNT1, TRPV3, TTC37, TTC7A, TYK2, UBA1, UBE2T, UHRF1, UNC119, UNC13D, UNC93B1, UNG, USB1, USP18, VAV1, VPS13B, VPS45, VTN, WAS, WDR1, WIPF1, WNT2B, WRAP53, XIAP, XRCC2, ZAP70, ZBTB24, ZCCHC8, ZNF341, ZNFX1

Table S2. Rare uncertain significance variants identified in the patient. The reference human genome used for the alignment phase was GRCh37/hg19. The American College of Medical Genetics and Genomics classification and GnomAD exome population frequency were based on the results consulted at march 2024.

Gene	Transcript	c.DNA	Protein	Zygosity	ACMG classification	GnomAD Exome*
COPA	NM_004371	c.1480G>A	p.Val494Ile	Heterozygous	VUS/LB	0.002%
ERBIN	NM_001253697	c.3886C>A	p.Leu1296Met	Heterozygous	VUS	N.D.
BACH2	NM_021813	c.1735A>G	p.Ile579Val	Heterozygous	VUS/LB	0.0203%

VUS: variant of uncertain significance; LB: likely benign; N.D.: not detected.

Figure S1. Immunophenotyping of T cells. Of the total number of lymphocytes, 94.3% are CD3+ T cells (represented with SSC-Linear and CD45+). Of these, an increase of CD8+ T cells (78% of the total CD3+) and 12.4% of double negative T cells (CD3+CD4-CD8-) were observed. Of the total CD8+ T cells, 82% expressed the CD57 marker.

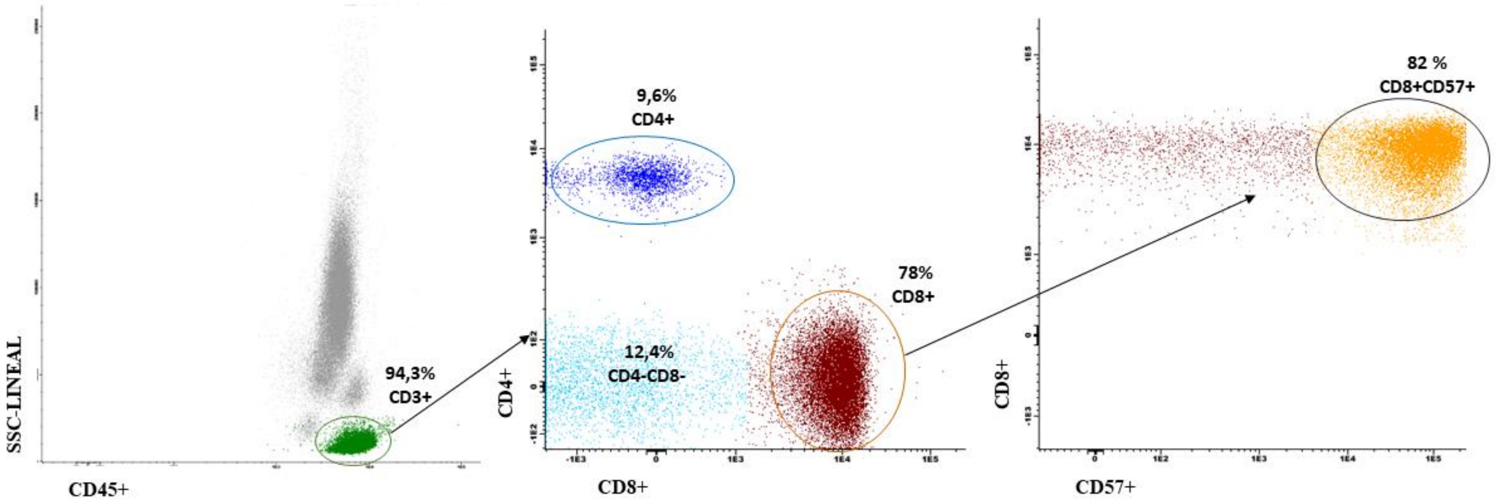

Supplement: Supplementary file 1 [file microorganisms-12-01078-s001.zip › microorganisms-2995672-supplementary.pdf]
